# Insights into the role of noradrenaline in effortful decisions

**DOI:** 10.1371/journal.pbio.3001545

**Published:** 2022-02-22

**Authors:** Kristin Kaduk, Fadila Hadj-Bouziane

**Affiliations:** 1 Decision and Awareness Group, Cognitive Neuroscience Laboratory, German Primate Center, Göttingen, Germany; 2 INSERM U1028, CNRS UMR5292, Neuroscience Research Center (CRNL), Impact Team, Lyon, France; 3 University of Lyon, Lyon, France

## Abstract

Because our energetic resources are limited, our brain needs to monitor effort to optimise behavioral choices and avoid fatigue. This Primer explores the implications of a recent PLOS Biology study showing that the activity of noradrenergic neurons in the locus coeruleus reflects the amount of effort exerted to face costly cognitive or physical challenges, a signal that might help promote behavioral adaptation depending on context and our needs.

## Teaser

To maintain homeostasis, our brain tracks the effort exerted during decision-making. A new study in *PLOS Biology* shows that the activity of noradrenergic neurons in the locus coeruleus (LC) reflects the effort exerted to face cognitive or physical challenges.

Every decision we make involves costs and benefits that we implicitly weigh. As our energetic resources are finite, the general consensus is that we naturally tend to minimize the costs while maximizing benefits (reward). Reward value is discounted by the effort invested and/or its perception, and so there is a natural tendency for animals and humans alike to avoid pursuing rewarding options that require effort.

It is thought that the brain computes a net score between costs and benefits in the medial prefrontal cortex [1], based on past experiences and potential future outcomes, to shape our decisions. To date, the neurobiology of reward is relatively well documented. We know, for example, that reward processing is under the influence of the dopaminergic system (DA) and the basal ganglia [2]. How about effort? For that matter, how do we quantify effort in the context of decision-making? Effort might refer to physical or mental activity needed to achieve a goal. While physical effort is quantified with metabolic expenditure related to biomechanical costs, mental or cognitive effort is more difficult to measure, although it also expends metabolic resources [3].

**Fig 1:**
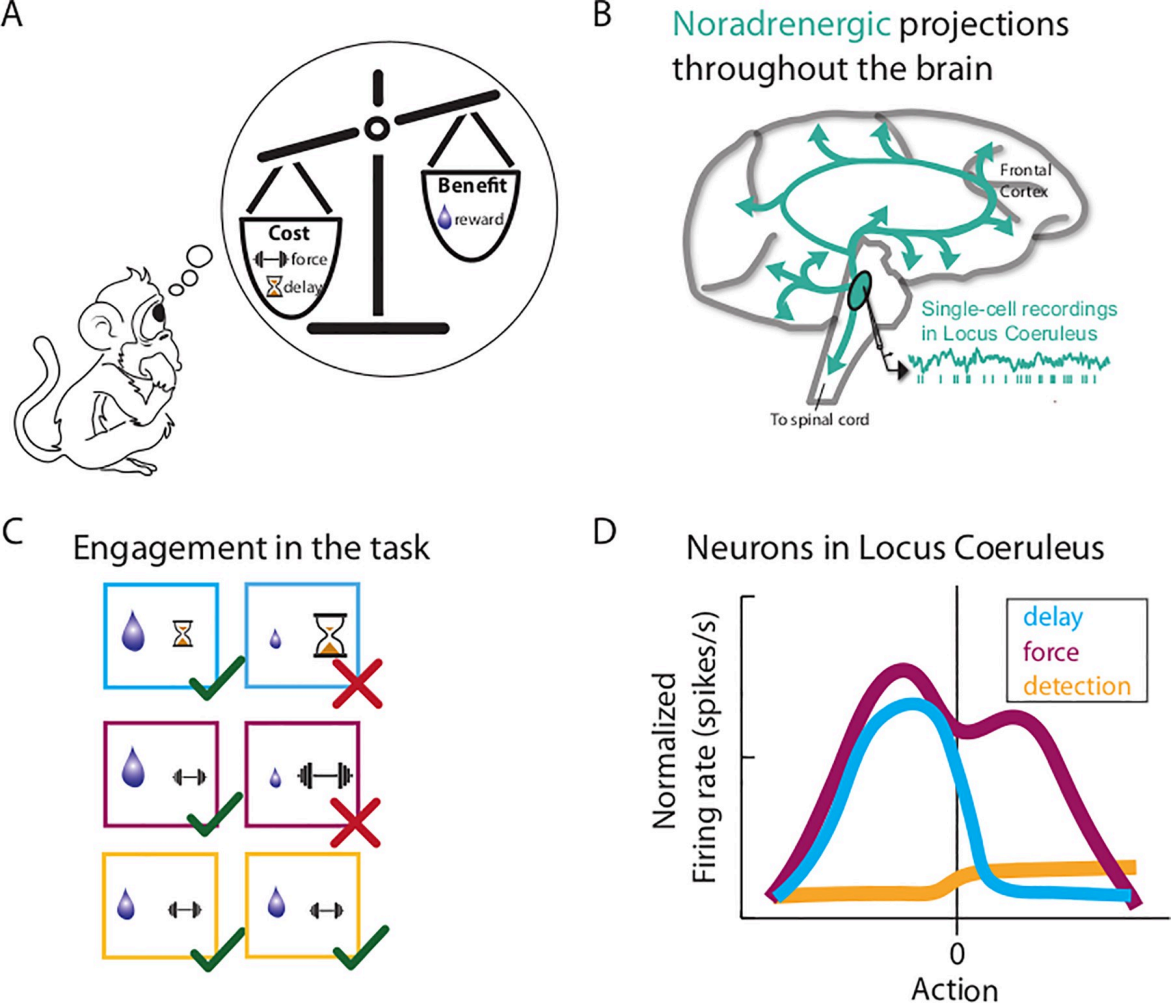
LC noradrenergic neurons encode effortful decisions. **(A)** Primates take decisions with the tendency to minimize costs while maximizing benefits. In the context of the experiment, costs involved either squeezing a grip more or less forcefully or waiting for more or less longer after releasing a lever while benefits involved different amounts of water. **(B)** Single-cell recordings were performed in the LC, a small nucleus in the brainstem that sends noradrenergic projections throughout the brain (in blue). **(C)** In 2 different tasks, the authors manipulated the level of physical effort animals had to exert on a grip (force, in purple) or the time delay after pressing a lever (delay, in blue) to earn various amounts of reward. In a third task (detection), animals had to apply a minimal force on a grip to receive a reward without any delay (in yellow). In the first 2 tasks, monkeys tended to avoid effortful options that entailed small amounts of reward, longer delays (in blue), or stronger physical forces (in purple), while in the detection task, there was no evidence for any bias. **(D)** The peristimulus time histogram symbolizes the mean firing rate of the population of LC neurons over time for the 3 tasks (delay, force, detection, in blue, purple, and yellow, respectively) aligned on the action onset. Right before the decision to act, LC neurons displayed a brief burst of activity only for the delay- and force-discounting tasks. Of note, the magnitude of this response was tightly related to the monkeys’ reaction time and reflected their willingness to perform the trial depending on its value and constraints. This activity was higher when the animal decided to engage in more demanding options (less reward, longer delay, or stronger grip) and was not modulated in the detection task. In addition, the activity also increased with more demanding physical effort in the force-discounting task. Together, these results suggest that the LC plays an important role in initiating and performing effortful actions. Given the widespread projections of the LC, these signals could be broadcasted toward other brain areas where increased NA availability could adjust sensory and cognitive processes to promote goal-directed behavior. LC, locus coeruleus; NA, noradrenaline.

Bouret and colleagues previously postulated that mobilizing resources to face challenges depends upon one deep brain structure, with about 15 000 neurons in primates, which contains the sole source of noradrenaline (NA): the locus coeruleus (LC) (Fig 1B). This structure is involved in key homeostatic and cognitive functions, including the regulation of the sleep-wake cycle, attention, memory, and the response to stress.

In this new study, Bornert and Bouret [4] highlight important properties of LC neurons compatible with the notion of subjective difficulty to overcome both physical and cognitive challenges.

They recorded LC neurons activity in monkeys engaged in different types of tasks where the level of reward (1, 2, or 4 drops of water) and effort (physical or cognitive) required to earn that reward were manipulated (Fig 1A). Specifically, to obtain a reward, in the force-discounting task, animals had to squeeze a pneumatic grip with 1 out of 3 possible levels of forces, while in the delay-discounting task, they had to exert an easy action (releasing a lever) and wait for a given period of time among 3 possible ones (from a couple of milliseconds to several minutes) before receiving the reward. In both tasks, the 9 [3 costs × 3 benefits] combinations were presented with equal probability. Importantly, in both tasks, the same trial was replayed until it was correctly performed by the monkey. This ensured that the animal does not get the option to avoid particular types of trials and instead completed them all.

Not surprisingly, the authors found that animals were more willing to forgo trials with stronger constraints (i.e., those requiring more physical force on the grip or waiting for longer periods of time) and/or smaller reward (Fig 1C). By contrast, when no delay and a minimal grip pressure was sufficient to earn a reward, the animals did not systematically avoid any options.

They found that LC neurons displayed a brief burst of activity when the animals decided to act and when they actually exerted the physical force (Fig 1D). Strikingly, the magnitude of LC neurons activity was higher in trials with lower reward or higher costs, suggesting that this activity before and after action reflects the effort that the animal is producing to face the challenge, be it cognitive or physical, to earn a reward. It should be noted that this pattern of activity was particularly well reflected at the population level rather than at the level of the individual neuron, suggesting a distributed coding of effort production throughout the LC.

What is particularly interesting in this study is the exploration of LC activity during cognitive and physical effort in relation to different levels of rewards. Plus, tracking the activity of such a deep and small nucleus in behaving monkeys is challenging and rare, yet critical given the exquisite temporal and spatial resolution it provides. How might these signals contribute to value-based decision-making? It is likely they are integrated over time within the medial prefrontal cortex and influence the seeking of better options should the effort associated with a current opportunity be too high. This hypothesis could not be explicitly tested in this work as the animals had only one choice at a time and needed to complete all trials sequentially. What if a choice between competing options, of the sort we often face, was provided? In the case of DA neurons, their activity either reflected the value of the future reward if only one option was offered or that of the best option when 2 of them were offered [5]. It would therefore be interesting to investigate if and how effort-related activity in NA neurons is influenced when various options are available.

The perspective of selectively silencing these neurons using optogenetics [6] might also prove extremely useful to determine their functional significance in tasks manipulating benefits and costs. How does this signal contribute to optimizing behavior while preserving us from chronic fatigue? A recent study suggests that the DA cares not only about reward but also integrates the willingness to exert mental effort [7], whereas the willingness to wait to avoid fatigue might be dependent on another important neuromodulator, serotonin [8]. Determining how these major neuromodulators interact is a fundamental and difficult question that remains unanswered. Last but not least, how do LC neurons respond when effort has an intrinsic reward value as shown for instance with wild mice exercising in running wheels under natural conditions, with no extrinsic reward [9]? The term “effort” is used in different contexts, to refer to task difficulty or internal self-control, and it is critical to define clearly what it entails to better capture its representation in the brain [10]. This effort is indeed necessary, as outcomes of this research are fundamental to develop strategies to overcome difficulties found in major pathologies featuring a lack of drive, such as in Parkinson disease or depression.

## Abbreviations

DA: dopaminergic system
LC: locus coeruleus
NA: noradrenaline
